# 

**Published:** 1904-12-17

**Authors:** 


### NOTICE TO CORRESPONDENTS.

All MSS., Letters, Books for Review, and other matters intended for the Editor should be addressed The Editor, "The Hospital," The Hospital Buildings, 28 & 29 Southampton Street, Strand, W.C.

The Editor cannot undertake to return rejected MS., even when accompanied by stamped directed envelope.

**Notice to Advertisers and Subscribers.**

All Advertisements, Orders for Copies of The Hospital, and Business Communications should be addressed to **THE MANAGER (Not to the Editor),** The Hospital, 28 & 29 Southampton Street, Strand, London, W.C.

The Cost of Subscription, post free, is as follows:—Per week, 3½d.; per quarter, 3s. 3d.; per half-year, 6s. 6d.; per annum, 13s. Foreign and Colonial:—Per annum, 19s. 6d., payable, in all cases, in advance.

**NOTICE.—Vol. XXXVI. of "The Hospital" (April to September 1904), in handsome cloth binding, is now on sale at the office of this Journal, price 10s. 6d. Cases for binding the Numbers contained in this Volume 2s. Index to Vol. XXXVI., 3½d., post free.**

**The Monthly part for December is now ready. Price 1s., by post, 1s. 3d.**

### BOOKS RECEIVED.

From the Author.

"The Construction of Hospitals for Consumption and other Infectious Diseases." By John W. Hayward, M.D.

C. Griffin and Co., Ltd.

"Official Year Book of Scientific and Learned Societies, 1904."

Cassell and Co., Limited.

"Under the Care of the Japanese War Office." By Ethel McCaul.

A. and C. Black.

"Who's Who, 1905."

"Who's Who Year Book, 1905."

"Englishwoman's Year Book, 1905."

### MEDICAL APPOINTMENTS.

Janet Campbell, M.B., B.S., Senior Resident Medical Officer to the Belgrave Hospital for Children, S.W. A. G. R. Cameron, M.B., B S.Durh., D.P.H.Camb., Medical Officer of Health for the Combined Sanitary District of West Sussex. T. Coogan, M.B., Ch.B.Vict., Resident Assistant Medical Officer of the Chorlton Union Workhouse. F. Dittmar, M.D.Glasg, D.P.H., Medical Inspector of the Local Government Board of Scotland. R. W. T. Ewart, M.B., C.M., Clinical Assistant to the Chelsea Hospital for Women. Donald G. Hall, M.A., M.D.Cantab., M.R.C.P.Lond., Assistant Physician to the Sussex County Hospital, Brighton Norman Maclaren, B.C.Cantab., F.R.C.S.Eng., Assistant Surgeon to the Cumberland Infirmary. S. G. Mostyn, M.B, B.Ch.Oxon., D.P.H.Camb., Medical Officer of Health for the County Borough of South Shields. Flora Murray, M.B., B.S., Junior Resident Medical Officer to the Belgrave Hospital for Children, S.W. L D. Parsons, M.B., Ch.B Edin., Assistant Colonial Surgeon and Port Medical Officer at Gibraltar. Wm. A. Reidy, L.R.C.P. and S.Edin., Medical Officer and Public Vaccinator for the Maesycwmmer District of the Newport Union. Mrs. Agnes F. Savill, M.A., M.D., M.R.C.P.I., Honorary Assistant Physician to St. John's Hospital for Diseases of the Skin, Leicester Square. W. F. Shaw, M.B., Ch.B.Vict, House Surgeon and Resident Obstetric Assistant Surgeon to the St. Mary's Hospital, Manchester. R. De Salis Stawell, M.B., B.C.Cantab., Physician to the Salop Infirmary. R. H. Steen, M.D.Lond., Medical Superintendent of the City of London Asylum, Stone. Arthur Tovey, M.R.C.S., L.R.C.P., Clinical Assistant at St. John's Hospital for Diseases of the Skin, Leicester Square. A. E. Townley, M.B., Ch.B.Vict., Medical Officer of Health, Oswaldtwistle Urban District. F. Wolverson, M.B., C.M.Glasg., District Medical Officer of the Walsall Union.

**"The Hospital" Institutional Library.**

**"Hospitals and Asylums of the World." 4 Vols., with a Portfolio of Plans, complete £12 12s.**

**"Burdett's Hospitals and Charities, 1904." 5s. net.**

**"Burdett's Official Nursing Directory." 8s. net.**

**"Cottage Hospitals, General, Fever, and Convalescent." 10s. 6d.**

**"Uniform System of Accounts for Hospitals and Public Institutions." New Edition, 4s. net.**

**"Hospital Expenditure: The Commissariat." 2s. 6d.**

**"A Legal Handbook for the Use of Hospital Authorities." 2s. 6d.**

**"The Cottage Hospital Case Book or Register of Patients." 10s. and 12s. 6d.**

**All these are published by the SCIENTIFIC PRESS, Ltd., and may be obtained through any bookseller, or direct from the publishers, 28 and 29 Southampton Street, Strand, London, W.C.**

### NURSING.

By Isabel Adams Hampton

(Formerly matron of the Johns Hopkins Hospital, Baltimore).

7/6 net; 7/10 post free.

### NURSING: Its Theory and Practice.

By Percy G. Lewis, M.D.

3/6 post free.

### SURGICAL WARD-WORK AND NURSING.

With particulars and Illustrations of the Instruments used in various operations.

By Alexander Miles, M.D.

3/6 net; 3/10 post free.

### A COMPLETE HANDBOOK OF MIDWIFERY.

Designed in accordance with the requirements of the Central Midwives' Board.

By Dr. J. K. Watson.

6/- net; 6/4 post free.

### SMALL TEXT-BOOKS.

**One Shilling each. Post Free.**

### PRACTICAL HINTS ON DISTRICT NURSING.

By Miss Amy Hughes.

### MENTAL NURSING.

By William Harding, M.D.Ed.

### THE MIDWIVES' POCKET-BOOK.

With Prefatory Chapter on the Midwives Bill.

By Miss Honnor Morten.

### GYNÆCOLOGICAL NURSING.

By G. A. Hawkins-Ambler, F.R.C.S.

### NOTES ON PHARMACY AND DISPENSING FOR NURSES.

By C. J. S. Thompson.

### FEVERS AND INFECTIOUS DISEASES.

By William Harding, M.D.

### THE NURSE IN HOT CLIMATES.

By Dr. E. Henderson.

1/1 post free.

### THE EXAMINATION OF THE URINE.

By J. K. Watson, M.D.

1/1 post free.

### MODERN NURSING IN PRIVATE PRACTICE.

By Sir William Bennett, K.C.V.O., F.R.C.S.

7d. post free.

### POCKET BOOK OF TEMPERATURE CHARTS.

Containing 17 Twelve-Hour, and 8 Four-Hour Charts, perforated for removal.

7d. post free.

*Send for New Catalogue of Nursing Manuals, Case-Books, &c., post free on application.*

**THE SCIENTIFIC PRESS, LTD.**, 28 & 29 Southampton St., Strand, London, W.C.

